# The influence of static magnetic fields on canine and equine mesenchymal stem cells derived from adipose tissue

**DOI:** 10.1007/s11626-013-9730-1

**Published:** 2014-01-30

**Authors:** Monika Marędziak, Krzysztof Marycz, Agnieszka Śmieszek, Daniel Lewandowski, Nezir Yaşar Toker

**Affiliations:** 1 Electron Microscopy Laboratory, University of Environmental and Life Sciences Wroclaw, Kozuchowska 5b, 51-631 Wroclaw, Poland; 2 Wroclaw Research Centre EIT+, Stablowicka 147, 54-066 Wroclaw, Poland; 3 Institute of Materials Science and Applied Mechanics, Wroclaw University of Technology, Smoluchowskiego 25, 50-372 Wroclaw, Poland; 4 Veteriner Fakültesi Biyokimya Anabilimdalı, İstanbul Üniversitesi, 34320 Istanbul, Turkey

**Keywords:** Magnetic field, Adipose-derived mesenchymal stem cells, Canine, Equine

## Abstract

The aim of this study was to evaluate the proliferation rate and morphological changes of adipose-derived mesenchymal stem cells of canine and equine origin (Eq- and CaAdMSC). Investigated cells were exposed to a static magnetic field (MF) with the intensity of 0.5 T. Proliferation activity of cells was determined with the Alamar Blue assay. Obtained results, normalized in respect to the control culture, showed that EqAdMSC exposed to MF maintained a high proliferation status, whereas proliferation activity of CaAdMSC cultured in the presence of MF was decreased. Estimations of population doubling time (PDT) also revealed that EqAdMSCs exposed to static MF achieved a twofold increase in the total number of cells in a shorter amount of time than the control culture. The PDT value obtained for investigated CaAdMSCs indicated that MF exposure resulted in the prolongation of population doubling time. Morphology of cells and cellular composition was investigated using a light inverted microscope and a fluorescent microscope. A scanning electron microscope was used for microvesicles (MVs) imaging. Obtained results showed that both cell types maintained fibroblastic morphology and did not reveal signs of apoptosis or necrosis. However, the MF had an influence on the MVs secretion. While EqAdMSCs propagated in the presence of MF were characterized by the abundant MVs presence, CaAdMSCs revealed poor secretory activity. The approach presented provides complex analysis, which enables one to determine changes in equine and canine cytophysiology.

## Introduction

The injuries of bones and cartilages are serious problems for veterinary medicine. Degenerative disorders of the musculoskeletal system are characterized by high occurrence, and affects both large and small animals (Butt 2002; Mohammed et al. 2007). To overcome this issue, many approaches are applied. Treatment options mainly involve the utilization of two classes of drugs; non-steroidal anti-inflammatory drugs (NSAIDs), and glucocorticosteroids (i.e., steroid drugs). These groups of drugs help to control pain and inflammation associated with tissue degeneration. However, administration of the aforementioned medications does not rebuild damaged tissue, but only temporarily improves the quality of an animal’s life (Luna et al. 2007; Carter et al. 1999). Therapies with autologous and/or allogeneic transplantations of mesenchymal stem cells (MSC) seem to be the solution for this problem. The goal of this approach is to trigger the healing process and restore tissue functionality. Mesenchymal stem cells are a unique population of adult cells with regenerative and immunomodulatory potential (Tuglu et al. 2010). Injections with MSCs are widely used for treating locomotive disorders and have shown many positive clinical results, confirmed with long-term follow-up studies (Marycz et al. 2012a; 2012b). This approach is considered safe and noninvasive, and directly impacts the degenerated tissue and inflammation site (Guest et al. 2008; Tugulu et al. 2010). Mesenchymal stem cells isolated from adipose tissue (AdMSCs) are often applied in the veterinary clinical orthopedic practice as well as in stem cell-based tissue engineering in combination with biomaterials synthesized with sol–gel method (Brehm et al. 2012; Marycz et al. 2013). It is proven that these cells are distinguished by high plasticity and have the ability to differentiate into cells of mesenchymal origin, like osteoblasts and chondroblasts. The AdMSCs produce factors that promote regeneration by stimulating new blood supply and activating resident stem cells in a paracrine manner (Puissant et al. 2005; Del Bue et al. 2008; Gonzalez et al. 2009). Additionally, soluble autocrine growth factors secreted by MSC increase their multi-potential character and are essential for self-renewal of the population (Zaragosi et al. 2006; Rider et al. 2008). Activated MSCs also produce small, spherical membrane fragments called microvesicles (MVs). As it was shown, MVs shed by adipose tissue-derived mesenchymal stem cells contain a variety of essential bioactive molecules including microRNA, mRNA, lipids, growth factors, and other proteins which improves regeneration of damaged tissues (Tetta et al. 2012). The regeneration process can also be accelerated by stimulation with a magnetic field (MF). Devices that generate static magnetic fields, for example, magnetic blankets, saddle pads, bands, or collars, are commonly utilized in veterinary orthopedics for pain treatment. Magnetotherapy has been an area of interest for many years. This method has been regarded as safe, effective, non-intrusive, and viable (Rosen 2003; Ganesan et al. 2009). Recent data claims that MF affects various cell functions such as cell growth, mitochondrial function, expression of genes, and differentiation and inhibition of apoptosis. As reported by Ganesan et al. and Hronik-Tupaj et al. (Ganesan et al. 2009; Hronik-Tupaj et al. 2011), magnetic field stimulation has the ability to enhance osteoblasts and chondroblasts differentiation and therefore, able to increase bone and cartilage formation. Moreover, the exposure to a magnetic field causes reduction of TNF-α and IL-6 levels of bone marrow cells and chondrocytes (Chang et al. 2003; Ciombor et al. 2003; De Mattei et al. 2011). In the veterinary clinical practice, autologous transplantations of AdMSCs could be combined with magnetotherapy. It seems that the mechanism of such an approach is synergistic and not yet fully understood.

The aim of this research was to investigate the influence of 0.5-T static magnetic field on equine and canine AdMSC’s cultures (equine origin (Eq)AdMSC and canine origin (Ca)AdMSC, respectively) over a 7-d exposure period. The affect of MF exposure was evaluated by means of observation of cells morphology, culture growth pattern, and evaluation of their proliferation and shedding activity.

## Materials and methods

### *Isolation of AdMSC*.

Adipose subcutaneous tissues were obtained from animals from the tail base area using standard surgical techniques. Permission from animal owners was obtained prior to the performance of procedure. Each tissue sample was placed into sterile Hank’s Balanced Salt Solution (HBSS). All stages of the procedure were conducted under aseptic conditions. Isolation was performed using well-established methods (Grzesiak et al. 2011). Briefly, the adipose tissues were washed extensively with HBSS containing 1% antibiotic-antimycotic solution (penicillin/streptomycin/amphotericin b). After removal of blood vessels, the tissues were cut into small pieces with surgical scissors. Samples were transferred into sterile tubes and digested with collagenase type I enzyme (1 mg/ml). Tissue samples were incubated for 30 min at 37°C. The tissue homogenate was centrifuged for 10 min at 1,200×*g* at room temperature. After centrifugation, the supernatant was removed while the cell pellet was re-suspended in growth media and seeded into a cell culture flask.

### *Cell culture*.

All cultures were propagated under aseptic conditions at the same growing and culturing conditions of 37°C, 5% CO_2_. The primary cultures were maintained in Dulbecco's Modified Eagle's Medium (DMEM)/Ham’s F12 medium, supplemented with 15% fetal bovine serum (FBS) and with 1% antibiotic-antimycotic solution. After 24 h of the primary cultures propagation, the medium was changed on DMEM with 4,500 mg/L of glucose, containing 10% FBS and 1% antibiotic-antimycotic solution. The medium was replaced every 2 d. When cultures achieved approximately 80% of confluence, cells were dissociated using trypsin solution (Life Technologies, Warsaw, Poland). Before the experiment, cultures were passaged four times. For the test, cells were placed into 24-well plates suspended in 500 μl of medium at a concentration of 35 × 10^3^ cells per well.

### *Magnetic exposure*.

**Figure 1.**
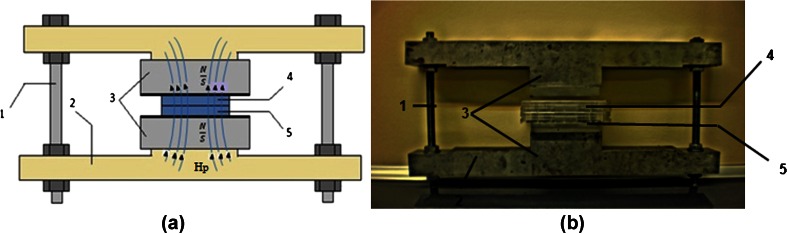
Exposure system: *a*, scheme, *b*, photograph. Description: *1*, bolts controlling the distance between magnets; *2*, external magnetic core; *3*, permanent magnets; *4* and *5*, culture dishes; *Hp* magnetic field vector; *N* and *S* magnetic poles.

A static magnetic field was produced by a pair of permanent neodymium magnets with a known magnetic polarization vector. The exposure system developed was previously described by Kaleta et al. (Kaleta et al. 2011). Culture dishes (127.89 × 85.6 × 19.69 mm) were placed in the gap between the two magnets (Fig. 1). Intensity of the obtained magnetic field was determined at about 0.5 T. Measurement of the magnetic field intensity was performed by hall sensor placed in the magnetic core gap. During the experiment, the magnetic field device with culture dishes was introduced to the CO_2_ incubator.

### *Cell proliferation assay*.

Cell viability was determined with the resazurin-based assay kit (Alamar Blue) and performed according to the manufacturer’s instructions. Viability of cells was estimated after 24, 48, 96, 120, 144, and 168 h. Absorbance levels of samples were measured using a microplate reader (SPECTROstar Nano, BMG LABTECH, Ortenberg, Germany) at a reference wavelength of 690 nm and subtracted from 600 nm. Proliferation factor (PF) was determined in relation to the control culture. Obtained values were presented as an arbitrary unit. Normalization of obtained data, with respect to the control culture, was used for determining whether the stimulation with a magnetic field resulted in an increase (PF >1) or decrease (PF <1) of AdMSC’s proliferation rate. The amount of cells was measured based on a standard curve performed during the test. Population doubling time was determined using a method previously published by Lee et al. (Lee et al. 2004), with support of a population doubling time online calculator (http://www.doubling-time.com/compute.php). Statistical analysis was performed using the Statistica 7.0 software (StatSoft, Inc., Statistica for Windows, Tulsa, OK). Six replications of the experiment were analyzed using statistical methods. The values were presented as mean SD. Differences between two variables were analyzed by non-parametric Mann-Whitney *U* test. The values of *p* less than 0.05 were considered to be significant.

### *Morphology evaluation*.

Morphology of cells, cellular composition, and culture growth pattern were evaluated with light, using a fluorescent and scanning electron microscope (SEM). Moreover, analysis with SEM allowed for MVs determination.

In order to perform observations, cells were fixed in 4% paraformaldehyde for 30 min at room temperature. For analysis of cellular composition, the fixation stage cells were later washed gently with HBSS. Washing of samples was performed between every step of the procedure. Before staining, cell membranes were permeabilized with 0.1% Triton X-100 for 15 min at room temperature. Cells were stained with atto-488-labeled phalloidin. Samples were incubated at room temperature, in the dark, for 30 min, after which, cells were counterstained with diamidino-2-phenylindole for 5 min. After triple washing, cells were observed under a light-inverted epifluorescent microscope (Zeiss, Axio Observer A.1). To observe cells with SEM, samples were washed with distilled water and dehydrated by passing through a graded series of ethanol-water mixtures (from 50% to 100%, every 10 min). Dried samples were than sprinkled with gold and placed in a microscope chamber. Morphology of the cells was evaluated using SE1 detector, at 10 kV of filament tension (SEM, Zeiss Evo LS 15, Carl Zeiss, Jena, Germany).

All reagents were purchased from Sigma Aldrich (Poznan, Poland) unless otherwise specified.

## Results

### *Proliferation of canine and equine AdMSCs in the static magnetic field*.

**Figure 2.**
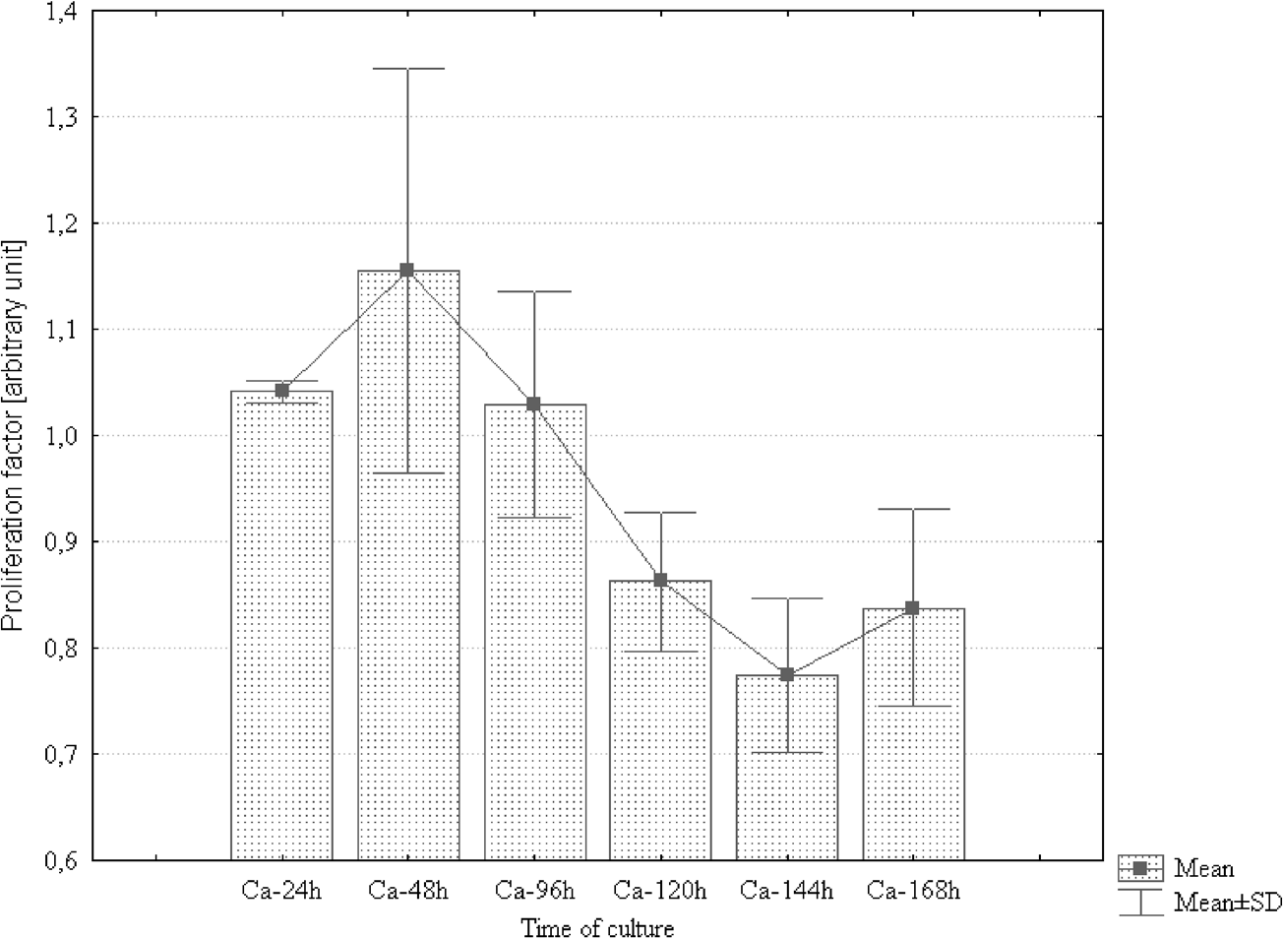
Distribution of proliferation ratio evaluated for canine AdMSCs exposed to magnetic field in selected time points.

**Figure 3.**
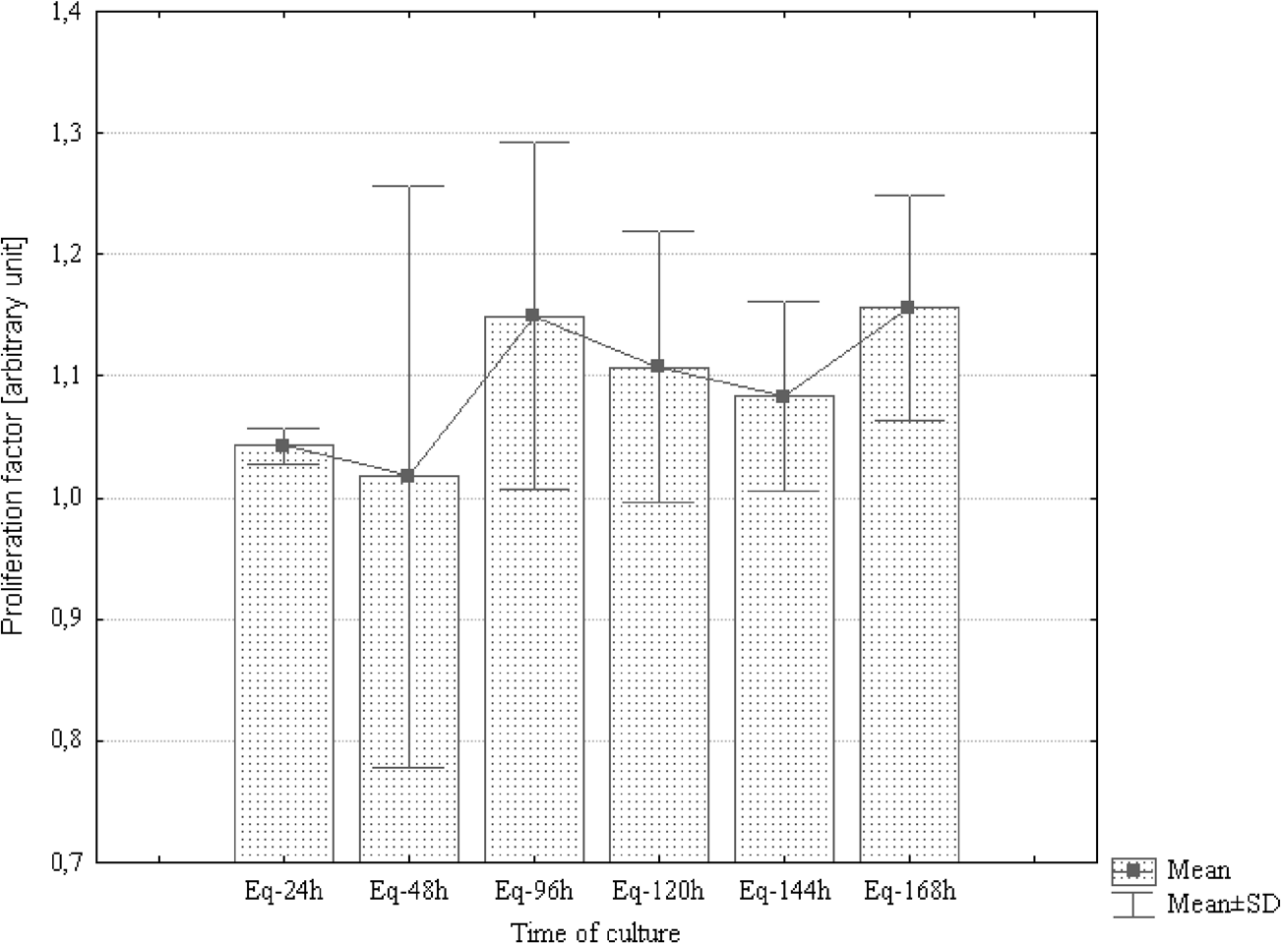
Distribution of proliferation ratio evaluated for equine AdMSCs exposed to magnetic field in selected time points. Marked differences were significant at *p* < 0.05.

The growth rate of AdMSCs was evaluated for 7 d, both in the presence as well as in the absence of static MF. Results obtained for the control culture were used for the determination of the relative proliferation ratio. Analysis revealed that the magnetic field significantly stimulated the proliferation activity of canine AdMSCs during the first 48 h of the experiment only. After 96 h, a rapid decrease in the proliferation rate was observed (Fig. 2). In contrast, the mean value of the proliferation ratio determined for equine AdMSCs culture exposed to static MF was higher than that of the controls throughout the entire experiment (Fig. 3).

**Figure 4.**
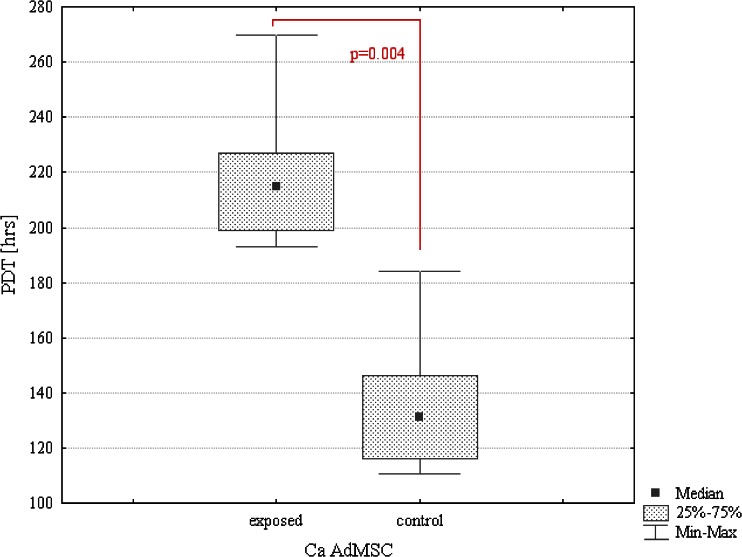
Comparison of PDT values. Statistical analysis of differences between MF-exposed and control CaAdMSCs’ cultures. Marked differences were significant at *p* < 0.05.

**Figure 5.**
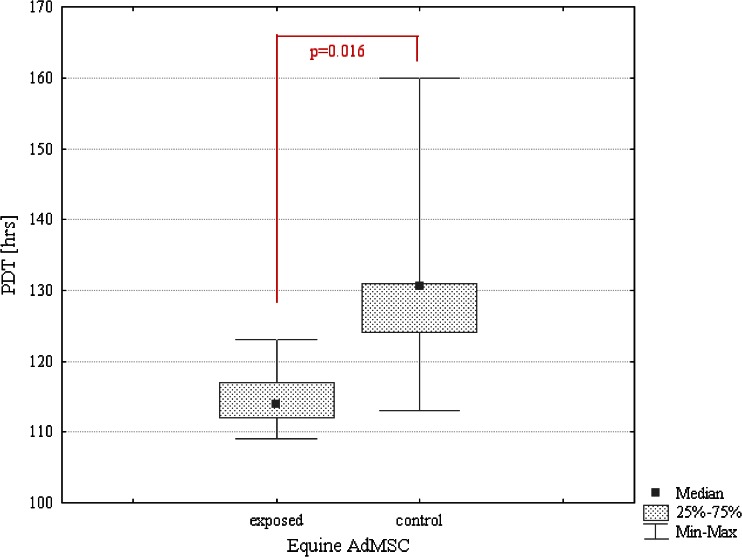
Comparison of PDT values. Statistical analysis of differences between MF-exposed and control EqAdMSCs’ cultures. Marked differences were significant at *p* < 0.05.

Moreover, exposure of canine AdMSCs to MF extended population doubling time about 40 %, whereas equine AdMSCs reached population doubling time sooner than in the control culture (Figs. 4 and 5). The differences in population-doubling times observed between control and exposed cultures were statistically significant (*p* ≤ 0.05).

### *Analysis of morphology and cellular composition*.

**Figure 6.**
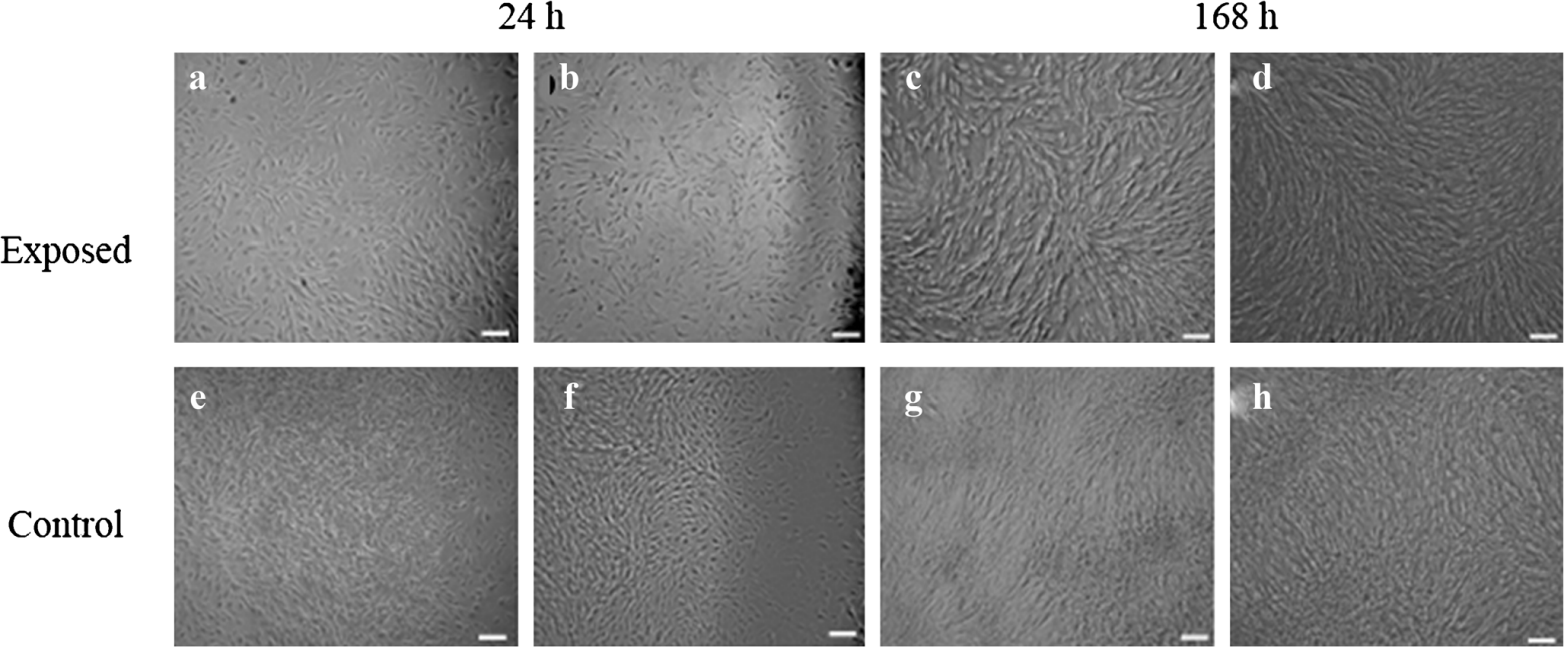
Morphology of canine AdMSC exposed to the magnetic field (*a*–*d*) and control culture (*e*–*h*). Evaluation performed after 24 (*a*–*b*, *e*–*f*) and 168-h propagation (*c*–*d*, *g*–*h*). Two area of culture well was observed, i.e., center (*a*, *f*, *c*, *d*) and figures show cell at the cells from the plate edge (*b*, *f*, *d*, *h*). Magnification ×50. *Scale bar* 200 μm.

**Figure 7.**
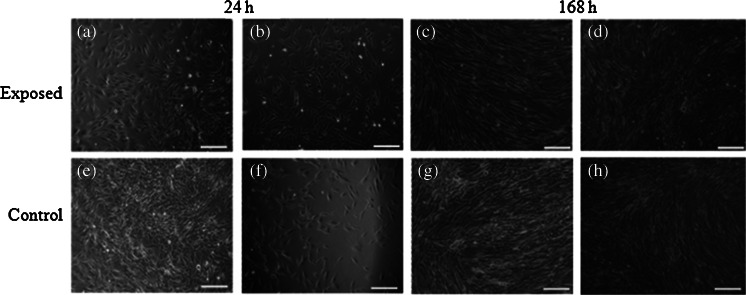
Morphology of canine AdMSC exposed to the magnetic field (*a*–*d*) and control culture (*e*–*h*). Evaluation performed after 24 (*a*–*b*, *e*–*f*) and 168-h propagation (*c*–*d*, *g*–*h*). Two area of culture well was observed, i.e., center (*a*, *f*, *c*, *d*) and figures show cell at the cells from the plate edge (*b*, *f*, *d*, *h*). Magnification ×100. *Scale bar* 200 μm.

**Figure 8.**
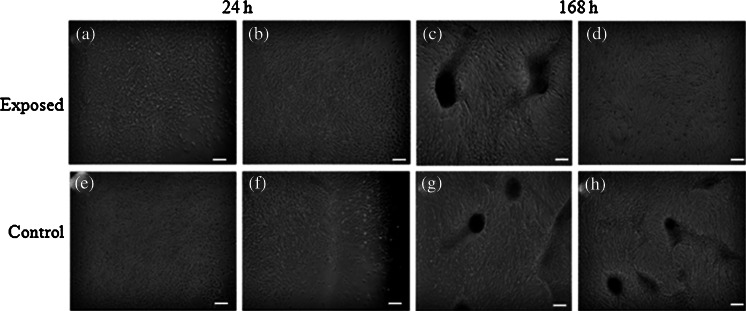
Morphology of equine AdMSC exposed to the magnetic field (*a*–*d*) and control culture (*e*–*h*). Evaluation performed after 24 (*a*–*b*, *e*–*f*) and 168-h propagation (*c*–*d*, *g*–*h*). Two areas of culture well were observed, i.e., center (*a*, *f*, *c*, *d*) and figures show cell at the cells from the plate edge (*b*, *f*, *d*, *h*). Magnification ×50. *Scale bar* 200 μm.

**Figure 9.**
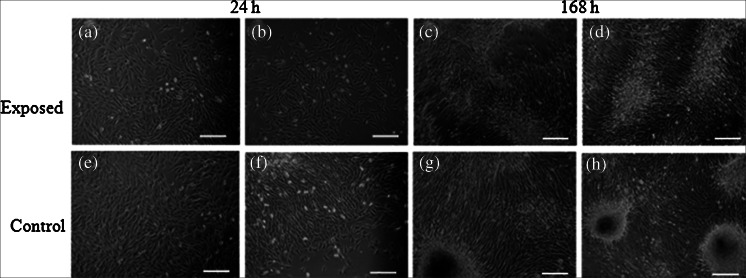
Morphology of equine AdMSC exposed to the magnetic field (*a*–*d*) and control culture (*e*–*h*). Evaluation performed after 24 (*a*–*b*, *e*–*f*) and 168-h propagation (*c*–*d*, *g*–*h*). Two areas of culture well were observed, i.e., center (*a*, *f*, *c*, *d*) and figures show cell at the cells from the plate edge (*b*, *f*, *d*, *h*). Magnification ×100. *Scale bar* 200 μm.

Canine, as well as equine cells, maintained typical fibroblast-like morphology. Microscopic observations after 24 h of culture revealed that both canine and equine cultures exposed to MF were evenly spread on the surface (Figs. 6, 7, 8, and 9*a*, *b*) in contrast to the control culture.

Both canine as well as equine AdMSCs in the control culture (not MF-exposed) were concentrated in the middle of the well and formed a multi-layer structure (Figs. 6, 7, 8, and 9*e*, *f*).

**Figure 10.**
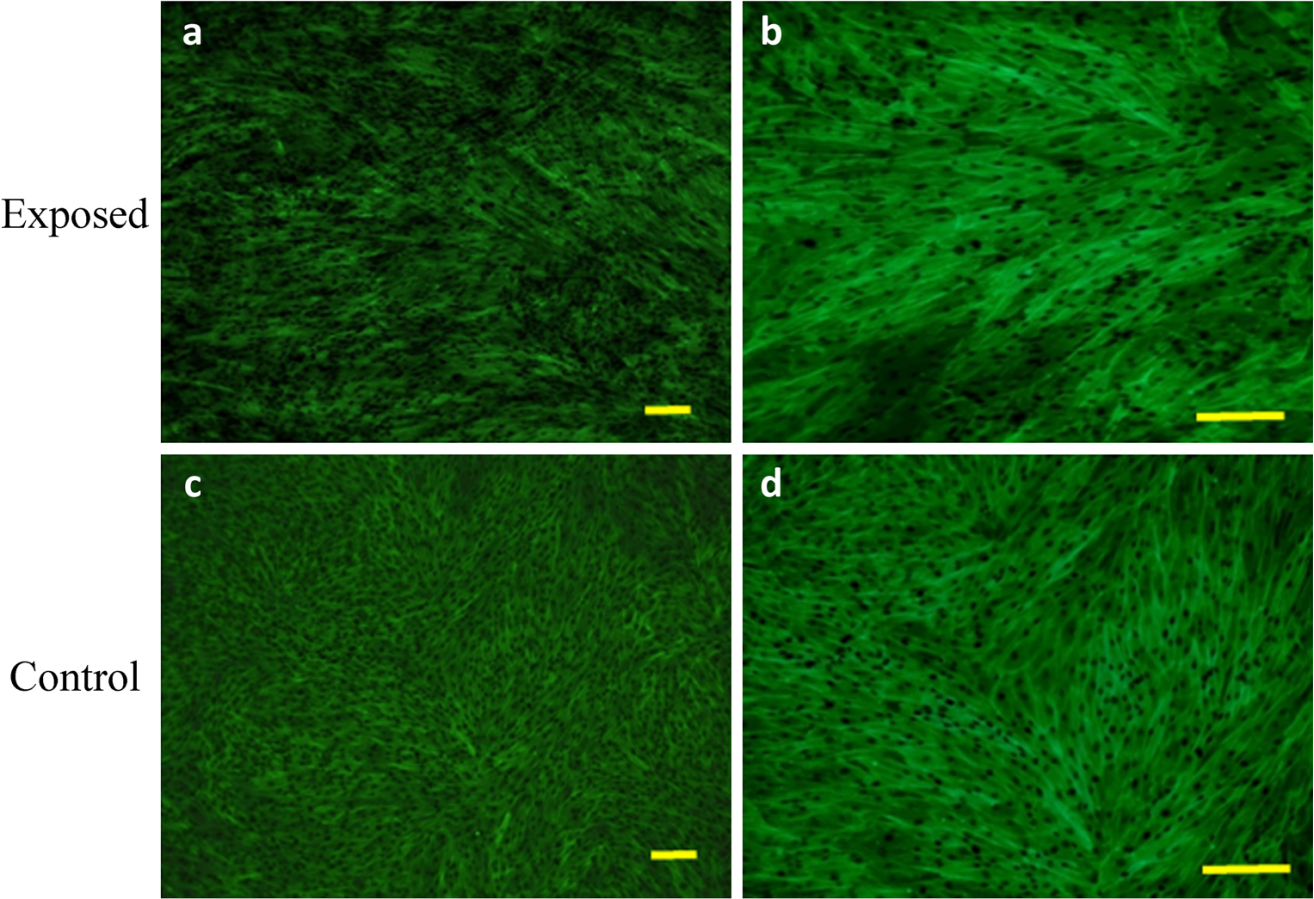
Morphology and cellular composition of canine AdMSCs. Cells exposed to the magnetic field are presented in *a* and *b*, whereas control culture in *c*, *d*. Staining was performed after 168 h of propagation. *Green*-stained elements f-actine filaments, *dark blue* nuclei. Magnification ×50 (*a*, *c*), ×100 (*b*, *d*). *Scale bar* 200 μm.

**Figure 11.**
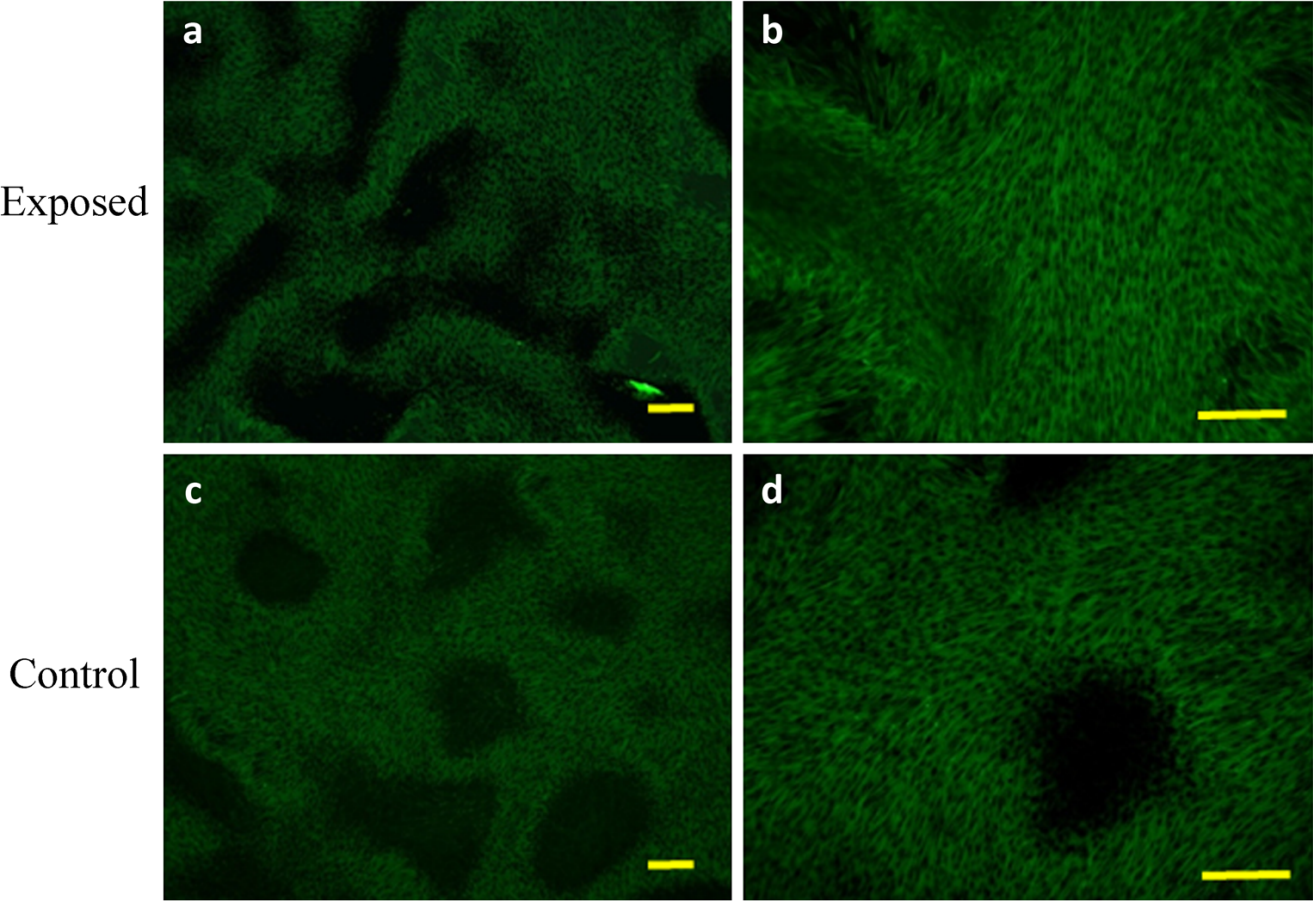
Morphology and cellular composition of equine AdMSCs. Cells exposed to the magnetic field are presented in *a* and *b*, whereas control culture in *c*, *d*. Staining was performed after 168 h of propagation. *Green*-stained elements f-actine filaments, *dark blue* nuclei. Magnification ×50 (*a*, *c*), ×100 (*b*, *d*). *Scale bar* 200 μm.

Evaluation of cell morphology and culture growth patterns performed after 168 h of incubation showed that cells closely adhere to each other and therefore adopted spindle-shape morphology. The confluence of the culture after 168 h was equal to approximately 90 % (Figs. 6, 7, 8, 9*c*, *d*, *g*, *h*, 10, and 11*a*–*d*). While EqAdMSCs were arranged in an orderly pattern both in an experimental and a control culture, CaAdMSCs exposed to MF had a directional growth pattern. It is also worth noting that exposure to the MF did not promote formation of apoptotic bodies.

### *Cell activity*   .

**Figure 12.**
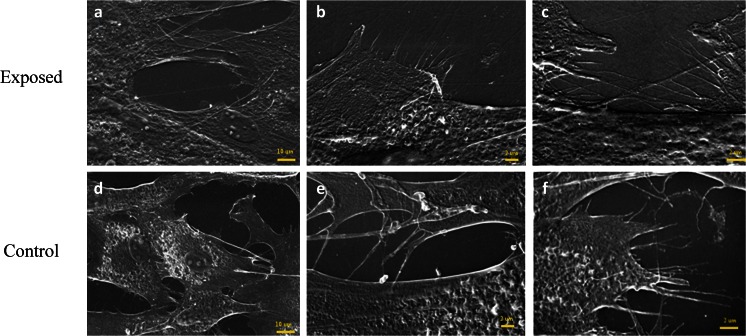
Microphotographs of canine AdMSCs cultured in the presence (*a*, *b*, *c*) and absence (*d*, *e*, *f*) of the magnetic field. Appropriate *scale bars* was indicated in the images.

**Figure 13.**
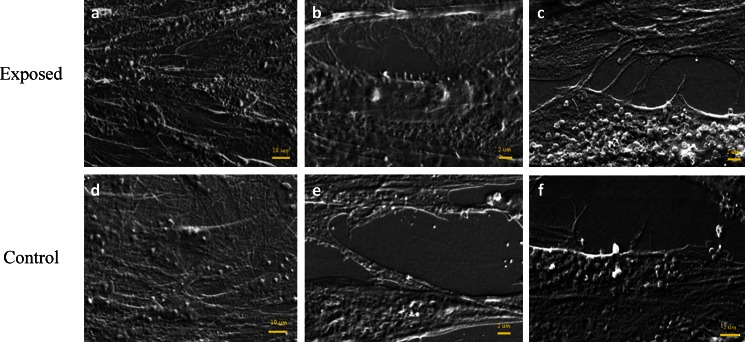
Microphotographs of equine AdMSCs cultured in the presence (*a*, *b*, *c*) and absence (*d*, *e*, *f*) of the magnetic field. Appropriate *scale bars* was indicated in the images.

Cell activity was determined based on the presence of synthesized microvesicles, produced by cells and deposited on their surface. Obtained results showed that treatment with the magnetic field stimulated equine AdMSCs and increased microvesicles shedding (Fig. 12). Moreover, this population of cells extensively developed intracellular connections via nanotubes as well as cytonemes. The magnetic field did not promote MVs secretion by canine AdMSCs (Fig. 13).

## Discussion

Magnetotherapy has been used in veterinary medicine to accelerate the regeneration of bone, cartilage, and tendons (Rosen 2003). This method is a form of physiotherapy and complementary to conventional clinical approaches, i.e., administration of NSAIDs and steroids. Despite the fact that this treatment is known from ancient times, current knowledge on biological mechanism has still not been clearly defined. Therefore, this subject, unfortunately, is one of the most easily misunderstood. The stimulating properties of MF have been investigated, both with the use of in vivo and in vitro models; however, obtained results still do not bring any ultimate conclusions (Rosen 2003; Yamamoto et al. 2003; Noriega-Luna et al. 2011; Ahmadianpour et al. 2013; Li et al. 2013). Another particularly interesting field of veterinary practice is therapies utilizing mesenchymal stem cells, especially those of adipose-tissue origin. Injections of mesenchymal stem cells are also often combined with magnetic field stimulation, in order to enhance the healing process of an animal. The regenerative potential of AdMSCs population is manifested by proliferative activity of the cells. This activity enables for self-renewal of MSCs population. Therefore, the main question is, Does MF affect the proliferation state? The answer to this query was one of the purposes of this paper.

In this study, we investigated the influence of a static magnetic field with the intensity equal to 0.5 T on the proliferation rate of canine and equine AdMSCs. Our results suggest that obtained MF affected the investigated populations of AdMSCs in a different manner. As a response to MF exposure, canine AdMSCs significantly reduced proliferation rate, whereas the proliferation activity of equine AdMSCs was enhanced. This result was confirmed with the comparative analysis of population doubling time values. As expected, canine AdMSCs cultured in the MF needed more time for population doubling than the culture not affected by MF, while equine AdMSCs in the exposure of a magnetic field had doubled its population quicker than cells in the control culture. Increased proliferative activity of cells is connected with high secretory activity. Our results confirm this effect. Equine AdMSCs with a high proliferation rate were also characterized by the presence of numerous MVs, unlike canine AdMSCs. Our results indicate the stimulating affects of MF on equine AdMSC. Increased numbers of MV and formation of nanotubes as well as cytonemes is connected with greater ability for cell-cell contact. Moreover, these structures are crucial for signaling, both in a paracrine as well as in an autocrine manner (Ratajczak et al. 2006; Tetta et al. 2012).

Despite significant changes in proliferation and secretory activity caused by MF exposure, we observed slight morphological changes manifested by the forming of multi-layer aggregates. This observation was common for both investigated populations of MSC; however, equine cells after magnetic field treatment showed that they closely adhered to each other, mainly on the center of the plate. After the first day of the cell propagation within the MF, in the single-cell evaluation, the shape of the cells did not modify. In the following days of exposure, cells attached themselves but no significant change in the shape of AdMSCs was observed. These findings were confirmed by Tenuzzo et al. (Tenuzzo et al. 2006) who reported that static MF did not significantly affect the thymocytes structure. These results contradict the research carried out with a static magnetic field, using other cell types and other field intensities, by Pacini et al., Xu et al., and Chionna et al., which reported considerable morphological modifications after MF treatment (Pacini et al. 2003; Chionna et al. 2005; Xu et al. 2008;). The reason for such differences may depend largely on different stress responses of cells exposed to static MF (Chionna et al. 2005). The MF enhanced the regularity of cells arranged in the culture. Cells exposed to MF had a characteristic directional growth pattern, noticeable especially in the early days of the culture at higher magnification.

## Conclusions

In summation, we conclude that the combination of stem cells and magnetic field treatment could have positive and synergistic affects in equine cures. This intensity of magnetic fields could be applied as a therapeutic strategy for equines, contrary to canines, where PDT and proliferation rates indicate that combined therapies may bring a poorer clinical affect than for horses. As a whole, the above results demonstrate that transplantations of AdMSCs could be combined with magnetotherapy and may have positive therapeutic results for horses.
